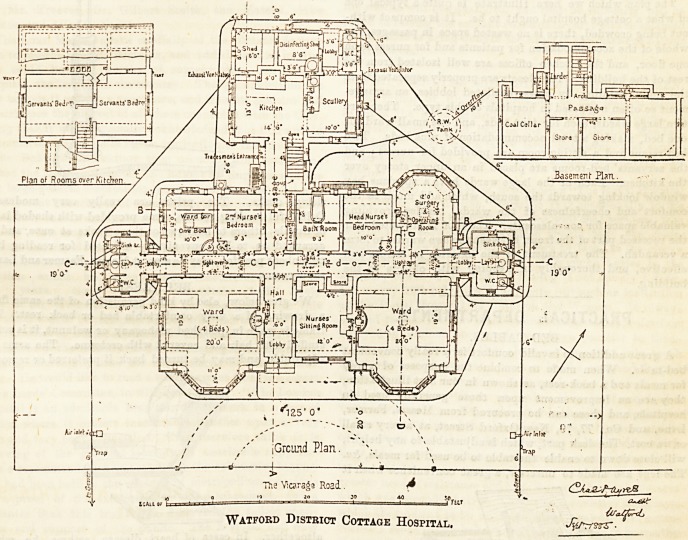# Watford Cottage Hospital

**Published:** 1893-05-20

**Authors:** 


### The Institutional Workshop.

### HOSPITAL CONSTRUCTION.

### WATFORD COTTAGE HOSPITAL.

We propose to give to-day plans of the Watford Cottage Hospital. A few weeks ago we went to Watford to inspect this institution, and found it closed owing to an outbreak of scarlet fever. We obtained admission, however, and went carefully over the building. It was undergoing a process of disinfection, and we were struck by the want of thoroughness apparent throughout the buildings. If there is one more potent disinfectant than another it is fresh air. Despite this, we found the windows closed, the atmosphere thick, and on opening some of the rooms the smell was most objectionable. We were unable to ascertain whether the chimneys had been swept, but our impression was that in all probability, unless someone with a sound knowledge of disinfection takes the matter in hand when patients are again admitted, there will almost necessarily be a second outbreak of fever. The infection of small-pox is probably the most difficult to get out of a building or ward, and next to that the infection of scarlet fever. In the course of a long experience we have come to the conclusion that if a ward is to be made absolutely safe from infection, it must be left vacant for at least a month, and during the whole of that time currents of fresh air should constantly pass through it. Of course, immediately after the patients have left the ward, it is desirable to remove everything from within the ward, which should then be disinfected by sulphur or some other process. After this has been done thoroughly the ward should be exposed to continuous currents fresh air for at least one month.

### The Watford Plan.

Mr. Charles Ayres, of Watford, the architect of the Watford Cottage Hospital containing nine beds, has produced a plan so compact and complete as to make it worthy of study. The building, which is most picturesque in outline, is of one storey, and the beds are placed in two wards of four beds each, with an isolation ward for one bed. The lavatories are disconnected from the wards, and operation and bath rooms are added. The kitchen is cut off from the main building by a passage, and the whole plan, as will be seen,

Watford District Cottage Hospital.

leaves little to be desired. The total cost of the buildings was, however, quite £1,800, making the cost per bed £200. This is a large outlay, and in addition to this expenditure £550 had to be provided to defray the cost of the site, £221 for furniture, and £31 for instruments, making the total cost upwards of £2,600, or nearly £300 per bed.

We are often asked the cost per bed of erecting cottage hospital buildings. Heretofore the pavilion plan has been so generally adopted that the initial outlay on certain cottage hospitals has been relatively enormous, and the working expenses afterwards are often in consequence so great as to dishearten the supporters of these institutions. The cottage hospitals at Stratford-on-Avon, at Darlington, and at Spalding are specially notable instances of this type, and the plans are worthy of study for this reason. Our experience convinces us that in all cases where the accommodation to be provided does not exceed ten beds, on the whole it can best be provided in an ordinary cottage, with slight and relatively inexpensive alterations. We should urge this view to the acceptance especially of wealthy people who may desire to give a cottage hospital building to the village or country district in which they take an especial interest. It is not wise or helpful to a district to present a small community with a new building beautiful in outline but expensive to maintain. Where this has been attempted, with the best intentions, by some wealthy landowner, it has not unfrequently happened that the cottage hospital has proved a burden too grievous to be borne, and thus the original founders by their generosity do, in effect, make the cottage hospital unpopular with the people of all classes, and so cause its existence to be imperilled, if the institution is not ultimately closed as too costly to be maintained by the limited population to the needs of which it was intended to minister.

The plan which we here illustrate is quite a typical one of what a cottage hospital ought to be. It is compact without being crowded, there is no wasted space in passages, the whole of the accommodation for patients and for nurses is on one floor, and the kitchen offices are well isolated from the rest of the building. The closets are properly separated from the main building by cross-ventilated lobbies, an arrangement so often neglected in hospitals of this type. There are two large wards, each for four beds, and one small ward for one bed, making a total accommodation of nine beds. Two bed-rooms and a sitting-room are provided for nurses; and the servants' bed-rooms are placed in an upper storey over the kitchen. Each of the large wards have a spacious bay window looking towards the south, which add much to the comfort and cheerfulness of the wards, besides providing valuable space for convalescents. The roof is projected over the recessed part of the front, between the two wards, to form a verandah. The treatment of the exterior is simple and effective, and thoroughly in keeping with objects of the building.

## Figures and Tables

**Figure f1:**